# The impact of warm-up with the volleyveilig approach on upper extremity function in female volleyball players

**DOI:** 10.1038/s41598-025-12730-z

**Published:** 2025-07-24

**Authors:** Adibeh Baharmast, Parisa Sedaghati, Somayeh Ahmadabadi

**Affiliations:** 1 Department of sports Injuries and Corrective Exercises, Faculty of Physical Education and Sports Sciences, University of Guilan, Rasht, Iran; 2 Department of Physical Education and Sports Sciences, Farhangian University, P.O. Box 14665-889, Tehran, Iran

**Keywords:** Injury prevention, Balance, Upper limb stability, Warm-up, Volleyball, Muscle, Tendons, Preventive medicine, Occupational health

## Abstract

This study aimed to assess the impact of the VolleyVeilig program on enhancing upper extremity performance in female volleyball players. While previous research has focused mainly on lower extremity injury prevention, there remains a significant lack of studies examining how structured warm-up routines can influence upper extremity performance in the sport. A quasi-experimental design with pre-test and post-test was used. Thirty female volleyball players, aged 16 to 21, were randomly divided into two groups: VolleyVeilig intervention group and traditional warm-up group. The VolleyVeilig warm-up consisted of light aerobic exercises, dynamic stretching, agility and reaction drills, as well as volleyball-specific skill drills conducted over six weeks. In contrast, the traditional warm-up included light aerobic activity, dynamic stretching, and volleyball-specific skill drills. Upper extremity performance indices including upper extremity balance and Davis tests were measured before and after the intervention. Statistical analyses, including the covariance analysis, and paired t-tests, were conducted using SPSS. Findings revealed a significant improvement in dynamic balance (Effect Size = 0.58, *p* = 0.001) and upper limb stability (Effect Size = 0.27, *p* = 0.001) in the VolleyVeilig group compared to the traditional warm-up. Additionally, significant enhancements were noted within the VolleyVeilig group from pre-test to post-test (*p* = 0.001). However, the small sample size and limited participant diversity may affect the generalizability of the findings. These findings indicate that the VolleyVeilig warm-up program effectively enhances dynamic balance and upper limb stability, potentially improving performance and reducing shoulder injuries. Coaches and sports therapists can integrate this 15–20-minute program into regular training sessions, incorporating aerobic exercises, dynamic stretching, and sport-specific drills, while adapting it to individual player needs for injury prevention such as shoulder impingement and rotator cuff strain, and performance optimization.

## Introduction

Volleyball, while considered a low-contact sport, still poses a risk for specific musculoskeletal injuries due to its repetitive and dynamic movements. One of the most commonly affected areas is the shoulder, which endures considerable mechanical stress during overhead actions like serving and spiking^1^. This repetitive strain can lead to overuse injuries such as rotator cuff tendinopathy, shoulder impingement syndrome, and labral tears^1–3^ especially when players have poor biomechanics or lack adequate muscular endurance and scapular stability^1^. Research by Naderza et al. highlights thaet simply having strong muscles is not enough to prevent fatigue from repetitive training. Their study indicates that even well-trained athletes can experience diminished performance and an increased risk of injury if they lack muscular endurance^4^. This suggests that muscular endurance is essential for maintaining optimal muscle function throughout prolonged training sessions^4^. Therefore, incorporating muscular endurance training along with strength development in injury prevention programs could significantly improve athletic performance and reduce the risk of fatigue-related injuries, particularly in the upper body^4,5^. To prevent these injuries, it is essential to concentrate on strengthening the rotator cuff and scapular stabilizers, enhancing technique, and implementing effective warm-up and recovery practices to reduce the risk of long-term damage^1^. While prior research has identified general risk factors associated with shoulder injuries in volleyball, there remains a paucity of evidence assessing the preventive efficacy of structured warm-up protocols that specifically focus on scapular and rotator cuff activation^6^. This deficiency underscores the necessity for controlled interventions aimed at evaluating the potential of targeted warm-up routines to effectively mitigate injury risk and enhance functional performance among volleyball players^7^.

In addition to shoulder injuries^8^ volleyball players are also at risk for injuries in the lower extremities, particularly the knees, ankles, and hips. The sport’s dynamic nature, characterized by frequent jumping, landing, and abrupt changes in direction, places considerable stress on the lower body. Activities such as repetitive jumping during spiking and blocking, along with rapid pivots and lateral movements in defensive play, can contribute to overuse injuries. Furthermore, the explosive actions inherent in volleyball may lead to muscle imbalances or insufficient joint stabilization in the lower extremities, increasing the likelihood of injuries^9–14^. Consequently, injury prevention is a vital concern in this sport, and implementing an effective warm-up routine is one strategy that can mitigate injury risk^15^. Researchers are actively working to develop warm-up protocols tailored to various sports. In this context, Mahmoudi et al. created a sports injury prevention program specifically for volleyball, which included a warm-up regimen featuring over 45 exercises focused on cardiovascular preparation and targeted injury prevention for the ankles, knees, and shoulders. This protocol has been proposed as an effective intervention for reducing the risk of injuries in volleyball, despite its length^5^. Given the challenges of using long and complex warm-up routines in regular training and competitions, it’s important to explore whether shorter, targeted warm-ups can still help prevent injuries. Focusing on areas that are prone to injury, like the shoulders and lower body, could be a practical and effective way to reduce the risk of injury in volleyball^16–19^. Recent studies underscore the significance of creating injury prevention programs and comprehending the biomechanics specific to volleyball in order to mitigate injury risk and improve performance. Most injuries among young volleyball players are musculoskeletal, which underscores the necessity for targeted prevention strategies^19^. As injury prevention programs have become integral to sports training, experts are increasingly advocating for sport-specific warm-up routines that address the unique demands and injury risks associated with each sport.

Protocols like FIFA 11 + have gained widespread recognition for their effectiveness in significantly reducing injuries among soccer players and referees^20,21^. In this regard, the VolleyVeilig program offers a tailored solution for volleyball, specifically addressing the injuries and movement patterns prevalent in the sport^16^. Research indicates that the FIFA 11 + injury prevention program led to a 35% reduction in injuries among female players and a 50% reduction in male and youth players^22^. Given the conceptual similarities between the FIFA 11 + program and the VolleyVeilig warm-up routine, it is plausible that implementing VolleyVeilig could also help prevent or mitigate injuries in recreational volleyball. The VolleyVeilig intervention comprises exercises focusing on core stability, strength, and balance, all selected to effectively lower the incidence of injuries^7,16^. Verhagen et al. found that the VolleyVeilig program was associated with a 30% reduction in acute and upper extremity injury rates, as well as a decrease in injury burden and severity among youth volleyball players^7^.

Further research in this field is crucial for creating evidence-based, sport-specific interventions that not only prepare athletes for competition but also reduce the risk of common injuries associated with volleyball^3^. Despite the prevalence of injuries, there is a notable lack of research on specialized injury prevention programs tailored to volleyball. This highlights the significance of targeted warm-up programs like VolleyVeilig, which cater to the specific physical demands of the sport, with a particular emphasis on dynamic balance, upper limb stability, and shoulder injury prevention^14,16^. Evaluating these specialized programs against traditional warm-ups can provide valuable insights for enhancing injury prevention strategies, ultimately leading to improved athlete performance and lower injury rates^16^. Given the limited comparative research on sport-specific warm-up programs in volleyball, particularly in the context of injury prevention, it is essential to assess the efficacy of specialized programs such as VolleyVeilig in relation to conventional warm-up routines. Investigating these differences can contribute to the identification of effective strategies for mitigating prevalent injuries, particularly those affecting the upper extremities. Consequently, further research in this area could aid in the development of targeted, evidence-based training programs, ultimately improving athletic performance and decreasing injury rates among volleyball players. Therefore, this study aims to investigate whether the VolleyVeilig warm-up program influences upper limb function differently than traditional methods in female volleyball players.

## Materials and methods

### Study design

This study is a quasi-experimental and applied research with a pre-test and post-test design, conducted under controlled and uniform conditions for participants in a safe environment at a sports club in Rasht city in 2023. The study obtained ethical approval from the Ethics Committee in Biomedical Research at Guilan University (Ethics code: IR.GUILAN.REC.1402.014).

### Participants

The statistical sample of this research comprised female volleyball players from Rasht City, aged between 16 and 20 years, with a minimum of 3 years of playing experience at the club level. The sample size was determined based on previous studies^17,23^ using G*Power software, with a test power of 0.80, an alpha level of 0.05, and an effect size of 0.50, resulting in a total of 30 participants. Thirty female volleyball players from Rasht City were purposefully selected based on inclusion criteria. This study randomly divided participants into two groups through simple randomization, with individuals assigned based on odd and even numbers. This resulted in the formation of the VolleyVeilig warm-up group, consisting of 15 participants, and the Traditional warm-up group, which also comprised 15 participants. Participants signed an informed consent form before the commencement of the research process.

### Inclusion criteria

Inclusion criteria for the study included being female, aged between 16 and 20 years, a minimum of 3 years of playing experience at the club level, not participating in any regular injury prevention programs during the previous competition season, no sports injury in at least the past 3 months, no physical therapy program (due to interference with research results), and no orthopedic problems^17^.

### Exclusion criteria

Exclusion criteria included unwillingness to continue participation in the study, the occurrence of an injury during the research process, and absence from more than two sessions in the training protocol^24^.

### Intervention group

#### Warm-up with the volleyveilig approach: a sport-specific injury prevention strategy for volleyball

The VolleyVeilig program is a warm-up regimen with an injury prevention approach specifically designed for the sport of volleyball, developed by Gouttebarge and colleagues^15,25^. This program utilizes an intervention mapping method and includes exercises focused on core stability, plyometrics, balance training, and volleyball-specific ball exercises. The program is conducted over a duration of six weeks, with three sessions per week. All exercises were carefully monitored and carried out in the direct presence of the researcher to ensure correct execution and compliance with the program’s guidelines.

The intervention, termed “VolleyVeilig^1^” was developed as an exercise-oriented warm-up program aimed at preventing or mitigating the incidence of both acute and overuse injuries, with a particular emphasis on shoulder, knee, and ankle injuries among volleyball players. This structured warm-up program is designed to last between 15 and 20 min and comprises several components: a preparatory cardiovascular warm-up lasting 2 to 3 min, core stability exercises for 2 to 3 min (e.g., the straight plank), targeted shoulder injury prevention exercises lasting 4 to 5 min (e.g., external rotation strength exercises utilizing resistance bands), knee injury prevention exercises for 4 to 5 min (e.g., squats), and ankle injury prevention exercises incorporated within the warm-up for 2 to 3 min (e.g., one-legged stances)^25,26^.

Table 1 presents a 6-week VolleyVeilig warm-up program designed for volleyball players, detailing the exercise content, intensity, and duration for each session.

**Table 1:** Six-week volleyveilig warm-up program for volleyball players.

Week	Component	Exercise example	Duration (min)	Intensity
1–2	Cardiovascular warm-up	Light jogging	2–3	Low to moderate
	Core stability	Straight plank	2–3	Moderate
	Shoulder injury prevention	External rotation with resistance bands	4–5	Moderate
	Knee injury prevention	Bodyweight squats	4–5	Moderate
	Ankle injury prevention	One-legged stance with eyes closed	2–3	Low to moderate
3–4	Cardiovascular warm-up	jogging	2–3	Moderate
	Core stability	Side planks	2–3	Moderate
	Shoulder injury prevention	Resistance band Y-T-W exercises	4–5	Moderate
	Knee injury prevention	squats	4–5	Moderate
	Ankle injury prevention	Single-leg	2–3	Moderate
5–6	Cardiovascular warm-up	jogging	2–3	High
	Core stability	Bird-dog exercise	2–3	Moderate
	Shoulder injury prevention	Overhead resistance band presses	4–5	Moderate
	Knee injury prevention	Lateral lunges	4–5	High
	Ankle injury prevention	Bosu ball balance exercises	2–3	Moderate

The website address for viewing the images of the VolleyVeilig exercises is provided in the footnote.

#### Traditional warming up for volleyball: control group

The traditional volleyball warm-up typically comprises three key elements: light aerobic exercises, such as jogging or jumping rope, to elevate heart rate and improve blood circulation; movements designed to target major muscle groups for enhanced flexibility and joint mobility; and sport-specific drills that focus on fundamental volleyball skills, including passing, setting, spiking, and serving, alongside movement drills. Participants in the control group were instructed to follow their standard warm-up routines and volleyball activities, which generally included running, both static and dynamic stretching, and concluded with ball-related warm-up exercises^27^. In the control group, the traditional warm-up program was carried out over a period of six weeks under the supervision of volleyball coaches, without any modifications to the regular warm-up content.

### Functional assessment tests

In this study, we utilized the Y Balance Test–Upper Quarter (YBT-UQ) and the Closed Kinetic Chain Upper Extremity Stability Test (CKCUEST) to assess the impact of the VolleyVeilig training program. Considering the aims of the current study and the nature of the VolleyVeilig program, which focuses on improving movement performance, dynamic balance, and upper extremity stability, particularly of the shoulders, the selection of the Y Balance Test–Upper Quarter (YBT-UQ) and the Closed Kinetic Chain Upper Extremity Stability Test (CKCUEST) is entirely appropriate and grounded in scientific evidence. These tests are recognized as reliable methods for evaluating dynamic balance, upper limb stability, and shoulder function^28^. Furthermore, these tests have the capacity to accurately and objectively measure key performance parameters that align with the preventive interventions of our program. In comparison to other available tests, these assessments allow for execution in controlled laboratory conditions and facilitate the collection of quantitative data, which offers advantages in terms of reliability and repeatability. Thus, the selection of these parameters has been made to ensure the scientific and practical standards of the conducted study. The YBT-UQ specifically measures athletes’ ability to control their upper body during movement^28,29^ which aligns closely with the primary objectives of the VolleyVeilig exercises. On the other hand, the CKCUEST assesses upper limb stability, muscular endurance, and neuromuscular control—critical components for preventing shoulder injuries among volleyball players^28,30,31^. Together, these assessments effectively reflect improvements in balance, stability, and reduced injury risk, showcasing the tangible benefits of the VolleyVeilig program.

#### Upper body Y balance test (YBT-UQ)

The YBT-UQ is a valid closed kinetic chain test for the upper extremity that can be utilized to assess unilateral upper extremity performance in athletes^32,33^. The Y Balance Test kit (Ghamat Pooyan, IN, Iran) on which the YBT-UQ is conducted consists of a standing platform to which three pieces of polyvinyl chloride (PVC) pipe are attached in the directions of anterior, posterior-medial, and posterior-lateral movements^34^. The posterior pipes are positioned at a 135-degree angle relative to the anterior pipe, and the angle between the posterior pipes is 90 degrees. Each pipe is marked with increments of 0.5 centimeters for measurement purposes. The participant pushes the target (the reaching indicator) along the pipe, standardizing the reach height (i.e., how far the reach is from the ground), and the target remains on the measuring tape during the test, enhancing accuracy in determining the reach distance. The variables of interest for this study included maximum normalized reach distances for each direction along with a composite reach distance. The greatest successful reach for each direction was utilized for analysis by each evaluator. Maximum reach distances were normalized based on the length of the participant’s upper extremity. To measure upper extremity length, the participant stood in an anatomical position while the researcher identified the C7 vertebra. After identifying C7, the researcher instructed the participant to elevate their right arm to shoulder height (90 degrees) (abduct). The tester then measured the distance from the spinous process of C7 to the tip of the furthest right middle finger (in centimeters) using a fabric measuring tape. The composite reach distance was calculated as the average of the greatest successful trials in each of the three normalized reach distances to analyze overall performance in the test. Average scores for the upper body Y balance test were reported as a percentage of limb length (%LL) for all reaching directions and the composite score^32,33^. Test–retest studies have consistently shown excellent reliability for the Upper Quarter YBalance Test, with ICC values ranging from 0.80 to 0.99 across various reach directions and inter-rater reliability reaching perfect agreement (ICC = 1.00)^32,35,36^.

#### Closed kinetic chain upper extremity stability test (CKCUEST)

This test is used to assess the dynamic stability or motor performance of the upper extremities. In this assessment, two strips of tape are placed parallel to each other on the ground, spaced 36 inches apart. The individual must assume a push-up position with their hands placed directly beneath their shoulders^37–39^. The participant is required to alternately reach each hand toward the opposite side and touch the corresponding strips of tape. This action must be completed within a time frame of 15 s, and ultimately, the number of times the individual successfully touches the opposite strips with both hands is recorded as the score^40^. The CKCUEST has demonstrated excellent reliability (ICC = 0.97) along with strong concurrent validity, correlating highly with grip strength (*r* ≈ 0.78–0.79) and isokinetic shoulder-rotation torque (0.87–0.94)^30,37,40^.

### Statistical analysis

For data analysis, SPSS version 26 was utilized, and the Shapiro-Wilk test was employed to assess the normality of the data. To compare the results between the VolleyVeilig warm-up group and the Traditional warm-up group, a covariance analysis was conducted. Additionally, to analyze the results between the pre-test and post-test within each group, a dependent t-test was used, while an independent t-test was applied to examine the homogeneity of groups concerning demographic characteristics. In the next section, the results derived from this methodology, including the analysis of participants’ demographic characteristics and statistical data analysis, are discussed in detail.

## Results

This section first addresses the demographic profile of the participants, such as age, gender, and other relevant factors, and then conducts statistical analyses to assess the program’s effects and compare the results across different groups.

The demographic characteristics of female volleyball players are presented in Table 2. The results comparing baseline characteristics between the VolleyVeilig warm-up group and the Traditional warm-up group, as assessed by the independent t-test, did not reveal any statistically significant differences; in other words, the groups were homogeneous in terms of demographic characteristics.

**Table 2:** Demographic characteristics of female volleyball players in the research groups.

Measurement index	Mean ± SD	Groups	T	P*
Age (yr)	1.95 ± 18.66	Control	2.45	0.21
	2.06 ± 16.88	Experimental		
height (cm)	0.04 ± 1.63	Control	− 1.01	0.31
	0.05 ± 1.65	Experimental		
Weight (kg)	11.56 ± 67.56	Control	0.93	0.36
	9.29 ± 64.00	Experimental		
BMI (k/m^2^)	4.65 ± 25.35	Control	1.37	0.18
	3.02 ± 23.38	Experimental		
Lower limb length (cm)	3.63 ± 86.20	Control	− 1.30	0.20
	3.47 ± 87.90	Experimental		
Upper limb length (cm)	3.36 ± 83.76	Control	− 0.84	0.40
	4.18 ± 84.93	Experimental		
Sports history (yr)	2.23 ± 8.13	Control	1.70	0.10
	1.57 ± 6.93	Experimental		

SD, standard deviation; cm, centimeter; kg, kilogram.

*Difference between the intervention and control groups (*p* < 0.05).

### Comparison of pre-test and post-test results for each of the warm-up programs

The comparison of research variables between the pre-test and post-test for each of the warm-up programs (VolleyVeilig warm-up and Traditional Warm-Up) was conducted separately using paired t-tests, with results reported in Table 2. According to the data in Table 2, the results of the paired t-test indicate that the VolleyVeilig warm-up program had a significant impact on balance variables in the medial direction (*p* = 0.001; ES = 1.63), inferior-lateral direction (*p* = 0.001; ES = 0.92), and superior-lateral direction (*p* = 0.001; ES = 0.69), as well as on the dynamic stability of the upper limbs (*p* = 0.001; ES = 1.32) (*p* < 0.01). However, in the Traditional warm-up group, no significant differences were observed between pre-test and post-test scores for balance and upper limb stability after 6 weeks (*p* ≥ 0.01). Table 3 illustrates the differences in mean balance and upper limb stability among participants before and after implementing the training protocol.

**Table 3:** Comparison of mean balance and upper limb stability before and after exercise Protocol.

Groups	Control group (*N* = 15)					Experimental group (*N* = 15)				
	Pre-test (Mean ± SD)	Post-test (Mean ± SD)	T	*P*	ES	Pre-test (Mean ± SD)	Post-test (Mean ± SD)	T	*P*	ES
YBT-UQ (medial) (%LL)	81.50 ± 6.35	83.90 ± 4.99	− 1.79	0.09	0.43	85.20 ± 4.90	94.43 ± 6.41	− 4.74	0.001*	1.63
YBT-UQ (inferolateral) (%LL)	65.70 ± 10.05	68.65 ± 9.93	− 1.70	0.11	0.29	72.93 ± 13.65	84.31 ± 10.47	− 4.74	0.001*	0.92
YBT-UQ (superolateral) (%LL)	36.80 ± 10.10	38.03 ± 9.99	− 1.18	0.25	0.12	41.96 ± 10.28	49.03 ± 10.16	− 4.74	0.001*	0.69
YBT-UQ (Overall) (%LL)	61.35 ± 7.63	63.52 ± 7.50	− 2.14	0.51	0.28	66.70 ± 8.40	75.92 ± 7.55	− 4.74	0.001*	1.15
Upper limb stability	16.63 ± 2.54	16.07 ± 4.69	0.31	0.75	0.15	18.50 ± 2.49	22.40 ± 3.40	− 7.44	0.001*	1.32

YBT-UQ, Upper Quarter Y Balance Test; %LL: percentage of limb length, ES: effect size.

*Difference between the pre-test and post-test in each group (*p* < 0.01).

### Comparison between the VolleyVeilig warm-up group and the control group with the Traditional warm-up program

The results of the analysis of covariance presented in Table 4 indicate that after controlling for the effect of the pre-test (as a covariate), there are significant differences in the performance results of the upper limbs, including balance and stability, in the post-test. Specifically, for balance variables in the medial direction (*p* = 0.001; ES = 0.42), inferior-lateral direction (*p* = 0.001; ES = 0.46), and superior-lateral direction (*p* = 0.001; ES = 0.32), as well as for dynamic stability of the upper limbs (*p* = 0.003; ES = 0.27), significant differences were observed between the VolleyVeilig warm-up group and the Traditional warm-up group (*p* < 0.01). The results indicate that the balance and stability of the upper limbs in the VolleyVeilig warm-up group were significantly better than those in the Traditional warm-up group.

**Table 4:** Comparison of balance and upper limb stability: ANCOVA results between groups.

Variables	Groups	Test stage	Mean*	F	df	*P*	Eta squared
YBT-UQ (medial) (%LL)	Control	Post-test	84.81	19.74	1	0.001**	0.42
	Experimental	Post-test	93.51				
YBT-UQ (inferolateral) (%LL)	Control	Post test	71.21	23.01	1	0.001**	0.46
	Experimental	Post-test	81.74				
YBT-UQ (superolateral) (%LL)	Control	Post test	40.28	12.68	1	0.001**	0.32
	Experimental	Post-test	46.77				
YBT-UQ (Overall) (%LL)	Control	Post-test	65.79	38.31	1	0.001**	0.58
	Experimental	Post-test	73.66				
Upper limb stability	Control	Post-test	17.00	10.46	1	0.003**	0.27
	Experimental	Post-test	21.47				

YBT-UQ, upper quarter Y balance test; %LL, percentage of limb length.

*Covariate factor = pre-test values.

**Difference between the experimental and control groups (*p* < 0.01).

## Discussion

The current study aimed to assess the effectiveness of a six-week volleyball-specific warm-up program, known as VolleyVeilig warm-up, in comparison to a Traditional warm-up program, focusing on upper limb performance, including dynamic balance and stability, in female volleyball players. Developed in 2017, the VolleyVeilig warm-up program was based on an analysis of movement patterns and injury-prone areas in volleyball players. Gouttebarge et al. investigated the impact of the VolleyVeilig warm-up program on injury reduction among volleyball players in studies conducted in 2017 and 2020. This program is notable for being one of the first randomized trials to evaluate the effectiveness of specialized warm-up interventions in preventing both acute injuries and overuse injuries in volleyball players. It includes targeted exercises for the shoulders and upper limbs, knees, and ankles, along with core stability exercises. Findings revealed that the VolleyVeilig warm-up program resulted in a 21% decrease in acute injuries^25,26^.

In this study, we examined the impact of the VolleyVeilig warm-up program on injury risk factors, specifically focusing on upper limb balance, stability, and core muscle endurance^25,26^. The findings revealed that the VolleyVeilig warm-up program significantly surpassed the Traditional warm-up group, which mainly utilized general warm-up exercises. This led to notable improvements in key areas, particularly in upper limb balance and stability among volleyball players. Consequently, the enhancement of these factors, as highlighted in earlier research, indicates that the VolleyVeilig warm-up program may positively affect performance and aid in injury prevention in volleyball.

Evert Verhagen and colleagues demonstrated in their study that the “VolleyVeilig” program, as a targeted warm-up intervention, significantly reduces the incidence of acute injuries and upper extremity injuries among adolescent volleyball players, while also improving the overall severity of injuries. These findings underscore the importance of employing structured and specialized warm-up interventions in the prevention of sports injuries and align with the results of several previous studies emphasizing the significance of preventive programs^41,7,42–44^. However, the degree of adherence to the complete execution of these warm-up programs may influence their effectiveness, highlighting the necessity of addressing practical aspects and the feasibility of implementing such programs in real training environments. Therefore, enhancing training methods and increasing commitment to the program could play a crucial role in improving its efficacy. These findings not only strengthen the scientific basis for utilizing targeted warm-up programs but also indicate the need for future studies to further investigate the factors influencing adherence and optimization of these programs. Additionally, they may emphasize the importance of designing and implementing specialized warm-up interventions to reduce upper extremity injuries in female volleyball players.

The VolleyVeilig program focuses on volleyball-specific movements, playing a crucial role in enhancing player performance and preventing injuries. It includes resistance and balance exercises that engage neuromuscular pathways essential for volleyball actions, thereby improving coordination between the brain and muscles, as well as boosting force production and reaction speed. Furthermore, balance and stability exercises enhance proprioception, which increases awareness of limb positioning in space, vital for improving balance and motor control, ultimately lowering the risk of injuries caused by uncoordinated movements^7^. The program also optimizes coordination between agonist and antagonist muscles, minimizing unnecessary co-activation of opposing muscles. This results in more efficient movements and reduced energy expenditure during volleyball-specific activities. Additionally, dynamic stretching and mobility exercises expand the shoulder joint’s range of motion, decreasing the risk of injuries such as tendonitis or rotator cuff tears. This comprehensive warm-up protocol is tailored to enhance motor performance and muscle stability, effectively lowering injury risks for volleyball players^7,26^. However, while current warm-up programs aim to prevent injuries, it can be challenging to persuade coaches and players to adopt them solely for this purpose unless they also demonstrate a positive and direct impact on performance^45^. As a result, researchers are developing comprehensive training programs integrated into warm-up routines that target both injury prevention and the enhancement of athletic performance^46^. Additionally, the findings of this study revealed that the VolleyVeilig warm-up program had a significant impact on balance and upper limb stability. These results are consistent with earlier research, such as that by Jha et al.^47^ which explored the effects of core stability training on upper limb performance, and Zarei et al.^23^ who investigated a shoulder injury prevention program’s influence on proprioception and dynamic stability in young athletes. Both studies reported notable improvements in upper limb performance and stability among volleyball players due to their respective training programs. Warm-up routines play a vital role in injury prevention, as highlighted by numerous studies examining their effectiveness in sports and the factors that influence optimal athletic performance and injury prevention^48^. Various warm-up programs have been developed specifically to prevent injuries, often tailored to the movement patterns associated with particular sports. For example, the FIFA 11 + program was created for soccer, while Wrestling + was designed for wrestling; both address common injury mechanisms in their respective sports and have demonstrated effectiveness in reducing athlete injuries. Similarly, both the VolleyVeilig and FIFA 11 + programs aim to minimize injuries and enhance athletic performance through structured exercises. They share a focus on improving balance, strength, and neuromuscular control, but differ in their specific applications. The FIFA 11 + program targets lower limb injuries in soccer, incorporating exercises that strengthen leg muscles, enhance balance, and include plyometric training. In contrast, VolleyVeilig is tailored for volleyball players, prioritizing upper body stability, shoulder strength, and dynamic balance—key elements for executing overhead movements such as spikes and serves. Consequently, while both programs effectively reduce injuries, VolleyVeilig’s specialized approach may offer a greater impact on preventing sport-specific injuries, particularly in the shoulders and upper limbs^25,26^. Additionally, the structured nature of both programs may explain the alignment between FIFA’s research findings and our study results^20,21^. The clear framework and consistent training incorporated into both programs contribute to improved athletic performance and injury reduction, potentially leading to similar outcomes in research across these areas^48^.

In this study, the control group followed a Traditional warm-up protocol, a widely used approach among volleyball players. This method generally emphasizes basic aerobic exercises, static stretching, and general movement patterns that prepare the body for physical activity. However, it lacks the sport-specific components necessary to target crucial areas such as dynamic balance and upper limb stability. Consequently, the Traditional warm-up did not adequately promote the neuromuscular adaptations needed to yield significant results in the tests conducted in this study. In contrast, the experimental group followed the VolleyVeilig warm-up protocol, which included exercises specifically designed to improve shoulder stability, dynamic balance, and neuromuscular control—essential elements for optimal volleyball performance. This sport-specific approach likely contributed to the notable improvements observed between the pre- and post-test results in the experimental group. The absence of targeted exercises in the control group’s Traditional warm-up may explain the lack of significant changes in their test outcomes.

The VolleyVeilig program, designed as a structured warm-up routine, has demonstrated significant effectiveness in reducing injuries and enhancing performance among volleyball players. To improve its implementation, it is advisable to customize the program for varying skill levels. For beginners, it is beneficial to lower the intensity and complexity of exercises, concentrating on developing fundamental movements and improving balance. In contrast, elite athletes should engage in more advanced activities, including plyometrics and specific muscle strengthening exercises, particularly targeting the shoulders and core. Furthermore, different program components can be integrated into various training phases, such as rehabilitation and specialized training, to prevent injuries and enhance overall athletic performance. Additional research involving a wider range of participants and diverse settings is essential to assess the applicability of these findings to other groups and sports. While the Y Balance Test for Upper Quarter (YBT-UQ)^32,33^ and the Closed Kinetic Chain Upper Extremity Stability Test (CKCUEST)^37–39^ are well-established as valid and reliable instruments for assessing upper extremity function, the present study did not incorporate a sensitivity analysis to examine the potential effects of procedural variations. It is important to recognize that factors such as limb positioning, tape spacing, and the manner in which instructions are delivered may significantly influence test outcomes. Therefore, future research should systematically investigate these variables to enhance the stability and reproducibility of findings. The inclusion of sensitivity analyses would not only strengthen methodological rigor but also bolster the generalizability of the results obtained.

### Limitations

One limitation of this study is that the sample consisted exclusively of female volleyball players, which may restrict the generalizability of the findings to other populations, including male athletes and participants from different sports. The uniformity of the sample could hinder the applicability of the results across diverse groups, as individuals of different genders or athletic backgrounds might respond differently to the training program. Consequently, further research involving a more varied participant pool is essential to determine whether the positive outcomes identified in this study can be extended to other demographics and sports. Other significant limitations include the small sample size and the brief duration of the study. The limited number of participants may impact the statistical power and generalizability of the results; therefore, future research should aim for larger sample sizes and multi-center trials to achieve more robust and widely applicable findings. A notable limitation of the present study is the lack of a sensitivity analysis to examine the potential influence of variations in key parameters on the observed outcomes. It is recommended that future research endeavors incorporate such analyses to evaluate the robustness and generalizability of the findings across diverse conditions. Additionally, the six-week duration may not adequately capture the long-term effects of injury prevention. It is advisable for future studies to incorporate longer follow-up periods, ranging from 6 months to a year, to evaluate the sustained impact of the program on injury prevention and overall performance enhancement.

## Conclusion

The VolleyVeilig warm-up program, with its coherent structure, can create a conducive environment for injury prevention and the enhancement of athletic performance among athletes, particularly volleyball players. Given that shoulder and upper limb injuries are among the most common injuries in volleyball, the observed improvement in upper limb performance following the implementation of this program indicates its potential in reducing the risk of such injuries. Therefore, incorporating these exercises into volleyball coaching training sessions is recommended. Additionally, this program holds significant potential for implementation in non-club settings, including schools and recreational leagues. Future research could examine the effectiveness of this program across different age groups and skill levels, as well as its long-term effects on injury reduction and athletic performance improvement.

## Data Availability

All data generated or analyzed during this study are included in this published article.

## Footnotes

1 https://www.volleyveilig.nl/selecteer-niveau-warming-up.
